# Development and validation of nomograms for predicting axillary non-SLN metastases in breast cancer patients with 1–2 positive sentinel lymph node macro-metastases: a retrospective analysis of two independent cohorts

**DOI:** 10.1186/s12885-021-08178-9

**Published:** 2021-04-26

**Authors:** Yang Yu, Zhijun Wang, Zhongyin Wei, Bofan Yu, Peng Shen, Yuan Yan, Wei You

**Affiliations:** 1 Department of Breast Surgery, Henan Provincial People’s Hospital, People’s Hospital of Zhengzhou University, People’s Hospital of Henan University, Zhengzhou, Henan Province China; 2 Department of Thyroid and Breast Surgery, Ruzhou First People’s Hospital, Ruzhou, Henan Province China; 3 Department of General Surgery, Maternal and Child Care Service Centre of Tanghe County, Nanyang, Henan Province China

**Keywords:** Nomogram, Axillary non-SLN metastases, Pathological features, Serum tumor markers

## Abstract

**Background:**

It is reported that appropriately 50% of early breast cancer patients with 1–2 positive sentinel lymph node (SLN) micro-metastases could not benefit from axillary lymph node dissection (ALND) or breast-conserving surgery with whole breast irradiation. However, whether patients with 1–2 positive SLN macro-metastases could benefit from ALND remains unknown. The aim of our study was to develop and validate nomograms for assessing axillary non-SLN metastases in patients with 1–2 positive SLN macro-metastases, using their pathological features alone or in combination with STMs.

**Methods:**

We retrospectively reviewed pathological features and STMs of 1150 early breast cancer patients from two independent cohorts. Best subset regression was used for feature selection and signature building. The risk score of axillary non-SLN metastases was calculated for each patient as a linear combination of selected predictors that were weighted by their respective coefficients.

**Results:**

The pathology-based nomogram possessed a strong discrimination ability for axillary non-SLN metastases, with an area under the receiver operating characteristic (ROC) curve (AUC) of 0.727 (95% CI: 0.682–0.771) in the primary cohort and 0.722 (95% CI: 0.653–0.792) in the validation cohort. The addition of CA 15–3 and CEA can significantly improve the performance of pathology-based nomogram in the primary cohort (AUC: 0.773 (0.732–0.815) vs. 0.727 (0.682–0.771), *P* < 0.001) and validation cohort (AUC: (0.777 (0.713–0.840) vs. 0.722 (0.653–0.792), *P* < 0.001). Decision curve analysis demonstrated that the nomograms were clinically useful.

**Conclusion:**

The nomograms based on pathological features can be used to identify axillary non-SLN metastases in breast cancer patients with 1–2 positive SLN. In addition, the combination of STMs and pathological features can identify patients with patients with axillary non-SLN metastases more accurately than pathological characteristics alone.

## Background

Breast cancer is the most common type of cancer in women and a leading cause of cancer-related death worldwide [1]. Sentinel lymph node biopsy (SLNB) is the standard treatment in early breast cancer patients with clinical negative axillary lymph node, and no further axillary treatment is required for sentinel lymph node (SLN) negative patients [2]. However, the optimal management of SLN positive patients remains controversial, since no more than half of patients have axillary non-SLN metastases when axillary lymph node dissection (ALND) is performed [3, 4]. In order to reduce unnecessary postoperative complications followed by ALND, breast-conserving surgery with whole breast irradiation has been recommended in patients with 1–2 positive SLN micro-metastases [5, 6]. However, whether patients with 1–2 positive SLN macro-metastases could benefit from breast conserving therapy and whole-breast radiotherapy remains controversial. Therefore, there is an urgent need to develop a nomogram for predicting the risk of non-SLN metastases in patients with 1–2 positive SLN macro-metastases.

Although pathology-based MSKCC breast nomogram [7] has been widely used to identify the patient’s individual risk of non-SLN metastases, its accuracy varies greatly among different populations (with an AUC ranges from 0.58 to 0.86) [8–10] and its application has not been validated in Chinese breast cancer patients. Wang et al. also has reported that tumor pathologic invasion size, number of positive SLNs and ALN status on imaging was associated with non-SLNs metastases in patients with 1–2 SLNs macro-metastases, but the included clinicopathological features and sample size are relatively small. Serum tumor markers (STMs) have been reported to be associated with the prognosis, recurrence and therapeutic effect of breast cancer [11, 12], whereas their predictive value for non-SLN metastases remains unknown. The aim of our study was to develop and validate nomograms for identifying patients at a high risk for axillary non-SLN metastases through their pathological features alone or in combination with STMs.

## Methods

### Study design and patient cohort

**Fig. 1:**
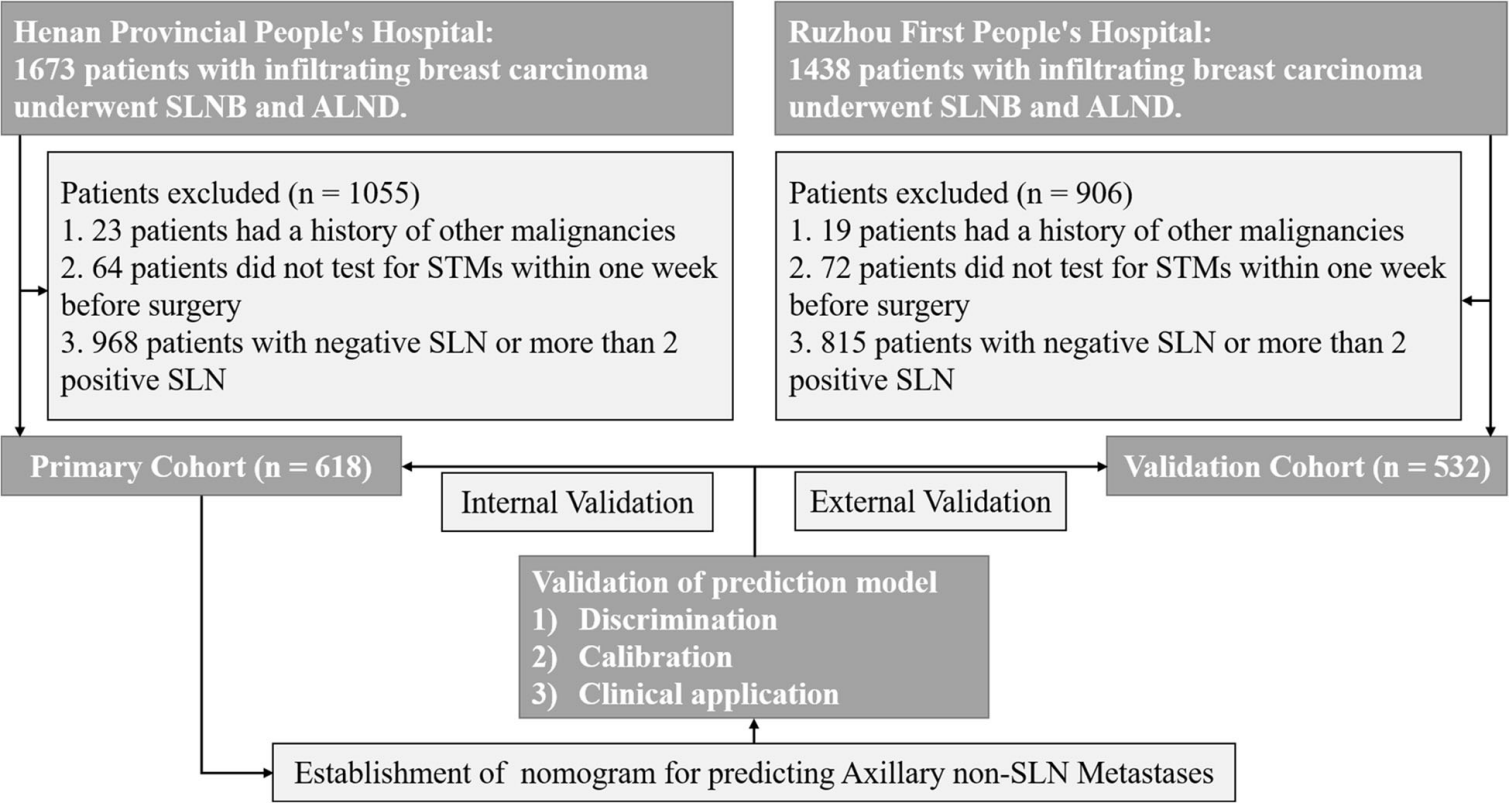
Flow diagram of establishing and validating nomograms for predicting axillary non-SLN metastases

The study was approved by the institutional ethics committee of People’s Hospital of Zhengzhou University (Henan Provincial People’s Hospital), and written informed consent was obtained from all participants in accordance with the Declaration of Helsinki. A primary cohort of 618 patients with histologically confirmed breast cancer was retrospectively analyzed between April 2016 and July 2020 at the Henan Provincial People’s Hospital (Henan, China). Inclusion criteria included the following: I) histologically confirmed infiltrating breast carcinoma; II) clinically negative axillary lymph node; III) pathologically confirmed 1–2 positive SLN macro-metastases; IV) completion of axillary lymph node dissection and histopathological assessment of dissected lymph nodes. Furthermore, an independent validation cohort of 532 patients was screened using the same criteria between October 2016 and November 2019 at Ruzhou First People’s Hospital (Henan, China). The diagram of establishing and validating our nomograms for predicting axillary non-SLN metastases in breast cancer patients with 1–2 positive SLN was shown in Fig. 1.

### Data collection

The pathological information of all eligible breast cancer patients was obtained from their medical records, including age, number of tumor lesions, tumor grade, histological type, T stage, number of positive SLN, number of negative SLN, lymphovascular invasion, estrogen receptor (ER), progesterone receptor (PR), human epidermal growth factor receptor 2 (HER-2) and Ki-67. Tumors displaying ≥10% nuclear-stained cells were considered to be tumor ER and PR positivity. HER-2/neu immunohistochemical staining was scored from 0 to 3+, 3+ was considered positive, and 0 or 1+ were considered negative. Fluorescence in situ hybridization tests were performed for patients with HER-2 scored as 2+. Tumor Ki-67 positivity corresponded to ≥14% nuclear-stained tumor cells. Serum samples obtained within 1 week before surgery were analyzed for each patient for carcinoembryonic antigen (CEA), carbohydrate antigen (CA) 125, and CA 15–3. Pathological cut-off levels were established as 5 ng/ml for CEA, 35 U/ml for CA 125, and 32 U/ml for CA 15–3.

### Nomograms building

The best subset regression was used to simplify prediction models when overfitting or multicollinearity occurred due to excessive amounts of variables. Logistic regression is one of the most commonly-used methods for establishing a prediction model to classify two groups of the population, and a nomogram is a practical tool to visualize the results of it, which we used to establish this prediction model. The AIC value for the final model was minimized with the fewest number of variables.

### Performance validation of nomograms

#### Discrimination

Discrimination ability was quantified by the area under the receiver operating characteristic (ROC) curve (AUC). AUC ranges from 0 to 1, with 1 indicating perfect concordance, 0.5 indicating no better concordance than chance, and 0 indicating perfect discordance.

#### Calibration

Calibration curves were plotted to assess the calibration of the nomogram [13], which consisted of two lines: one was a 45- degree reference line, and the other line represented the actual line. The interval between the two lines reflected the accuracy of the nomogram. Hosmer-Lemeshow test was used to evaluate the calibration of prediction model and a significant test statistic implies that the model does not calibrate perfectly.

#### Clinical usefulness

Decision curve analysis was conducted to determine the clinical usefulness of the nomograms via quantifying the net benefits at different threshold probabilities in the primary and validation cohorts [14, 15].

### Statistical analysis

Continuous data with normal distribution were expressed as mean (SD), while discrete variables were expressed as count (%). The alpha-level was set to 0.05, and statistical significance levels were all two sided. A *P* value < 0.05 was considered statistically significant. The continuous variables were transformed into binary variables by applying inflexion points of ROC curves as the cut-offs. Differences in continuous data between patients with and without axillary non-SLN metastases were analyzed using student’s t-tests. Chi-square tests or Fisher exact tests were used to examine the association of categorical data between these two groups. Statistical analysis was conducted with STATA 15.0 (Stata Corp, Texas, USA) and Rstudio software (Version 4.0.2, https://www.R-project.org).

## Results

### Clinical characteristics

In total, 1150 well-documented patients were recruited. The demographics and clinic features of these patients are shown in Table 1. Among the 1150 patients, A total of 618 patients (age range from 27 to 86 years) in the primary cohort and 532 patients in the validation cohort (age range from 26 to 89 years) met the inclusion criterion. There was no significant difference in the incidence of axillary non-SLN metastases between the primary and validation cohorts (29.5% vs. 30.6%, *P* = 0.659).

**Table 1:** The clinical characteristics of eligible breast cancer patients in the primary and validation cohort

	Training Cohort			Validation Cohort		
Clinical Features	non-SLN (Pos) (*n* = 182)	non-SLN (Neg) (*n* = 436)	*P* value	non-SLN (Pos) (*n* = 163)	non-SLN (Neg) (*n* = 369)	*P* value
Age (years)	53.9 ± 10.3	58.2 ± 12.7	0.216	54.4 ± 13.1	57.5 ± 12.9	0.326
No. of tumor lesions			< 0.001			< 0.001
Single	166 (91.2%)	425 (97.5%)		149 (91.4%)	363 (98.4%)	
Multiple	16 (8.8%)	11 (2.5%)		14 (8.6%)	6 (1.6%)	
Tumor grade			0.012			< 0.001
G1	12 (6.6%)	54 (12.4%)		9 (5.5%)	57 (15.4%)	
G2/G3	170 (93.4%)	382 (87.6%)		154 (94.5%)	312 (84.6%)	
Histological type			0.897			0.912
IDC	171 (94.0%)	407 (93.3%)		146 (89.6%)	334 (90.5%)	
Other	11 (6.0%)	28 (6.7%)		17 (10.4%)	35 (9.5%)	
T stage			0.037			0.082
T1/T2	93 (51.1%)	262 (60.1%)		87 (53.4%)	219 (59.4%)	
T3/T4	89 (48.9%)	174 (39.9%)		76 (46.6%)	150 (40.6%)	
No. of positive SLN			< 0.001			< 0.001
1	118 (64.8%)	325 (74.5%)		75 (46.0%)	314 (85.1%)	
2	64 (35.2%)	111 (25.5%)		88 (54.0%)	55 (14.9%)	
No. of negative SLN						
0	31 (17.0%)	17 (3.9%)	< 0.001	23 (14.1%)	19 (5.2%)	< 0.001
1	55 (30.2%)	68 (15.6%)		45 (27.6%)	54 (14.6%)	
≥2	96 (52.8%)	351 (80.5%)		95 (58.3%)	296 (80.2%)	
LVI			< 0.001			0.001
No	128 (70.3%)	353 (81.0%)		114 (69.9%)	287 (77.8%)	
Yes	54 (29.7%)	83 (19.0%)		49 (30.1%)	82 (22.2%)	
ER			0.236			0.352
Negative	51 (28.0%)	132 (30.3%)		49 (30.1%)	122 (33.1%)	
Positive	131 (72.0%)	304 (69.7%)		114 (69.9%)	247 (66.9%)	
PR			0.593			0.751
Negative	52 (28.6%)	138 (31.7%)		37 (22.7%)	97 (26.3%)	
Positive	130 (71.4%)	298 (68.3%)		126 (77.3%)	272 (73.7%)	
HER-2/neu			0.683			0.448
Negative	37 (20.3%)	83 (19.0%)		32 (19.6%)	81 (22.0%)	
Positive	145 (79.7%)	353 (81.0%)		131 (80.4%)	288 (78.0%)	
Ki-67			0.912			0.836
< 14	46 (25.3%)	110 (25.2%)		41 (25.2%)	89 (24.1%)	
≥14	136 (74.7%)	326 (74.8%)		122 (74.8%)	280 (75.9%)	
CA 125			0.839			0.910
Negative	164 (90.1%)	387 (88.8%)		139 (85.3%)	329 (89.2%)	
Positive	18 (9.9%)	49 (11.2%)		24 (14.7%)	40 (10.8%)	
CA 15–3			< 0.001			< 0.001
Negative	126 (69.2%)	416 (95.4%)		122 (74.8%)	339 (91.9%)	
Positive	56 (30.8%)	20 (4.6%)		41 (25.2%)	30 (8.1%)	
CEA			< 0.001			< 0.001
Negative	159 (87.4%)	414 (95.0%)		138 (84.7%)	350 (94.9%)	
Positive	23 (12.6%)	22 (5.0%)		25 (15.3%)	19 (5.1%)	

Abbreviations: *non-SLN (Pos)* positive non-sentinel lymph node, *non-SLN (Neg)* negative non-sentinel lymph node, *IDC* infiltrating ductal carcinoma, *SLN* sentinel lymph node, *LVI* lymphovascular invasion, *ER* estrogen receptor, *PR* progesterone receptor, *HER-2* human epidermal growth factor receptor 2, *CA* carbohydrate antigen, *CEA* carcinoembryonic antigen

### Clinicopathological features selection and nomogram building

**Fig. 2:**
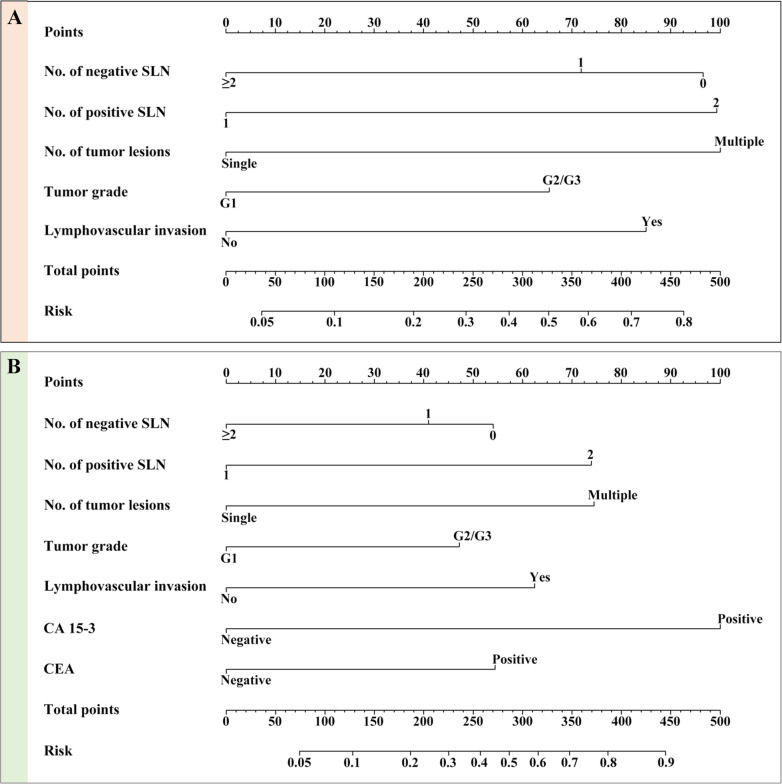
The nomograms for predicting the probability of axillary non-SLN metastases in breast cancer patients from the training cohort. **a** Pathology-based nomogram; **b** The combined nomogram

Among the twelve clinicopathological features in the primary cohort, five variables were finally selected as predictive factors to develop prediction model, including number of negative SLN, number of positive SLN, number of tumor lesions, tumor grade and lymphovascular invasion (Table 2). Using the regression coefficients of multivariate logistic regression models to weight each feature in our models, we developed a risk score formula to predict axillary non-SLN metastases: risk score = − 1.298 + 1.014 (if multifocal tumor) + 0.664 (if high grade tumor (G2/G3)) + 0.862 (if lymphovascular invasion is positive) + 1.342 (if number of positive SLN = 2) + (0.979, if number of negative SLN = 1; 0.729, if number of negative SLN = 0). Predicted risk = 1/(1 + e^−risk score^). To provide clinicians with a quantitative method for predicting the individual probability of axillary non-SLN metastases, we built a nomogram based on selected clinicopathological features (Fig. 2a).

**Table 2:** Risk factors for axillary non-SLN metastases in breast cancer patients with 1–2 positive sentinel lymph node

	Model 1			Model 2		
Intercept and variable	β	95% OR	*P* value	β	95% OR	*P* value
Intercept	−1.298		0.003	−1.168		0.011
No. of tumor lesions	1.014	2.757 (1.806 to 4.210)	< 0.001	1.034	2.811 (1.812 to 4.361)	< 0.001
Tumor grade	0.664	1.942 (1.246 to 3.027)	0.003	0.655	1.925 (1.217 to 3.045)	0.005
LVI	0.862	2.369 (1.574 to 3.565)	< 0.001	0.866	2.378 (1.558 to 3.630)	< 0.001
No. of positive SLN	1.342	2.740 (1.590 to 4.717)	< 0.001	1.376	2.786 (1.595 to 4.878)	< 0.001
No. of negative SLN						
≥ 2	reference			reference		
1	0.979	2.662 (1.393 to 5.087)	0.003	0.750	2.117 (1.085 to 4.130)	0.028
0	0.729	2.072 (1.099 to 3.907)	0.024	0.568	1.765 (0.919 to 3.390)	0.088
CA 15–3	NA	NA	NA	1.388	4.006 (2.330 to 6.887)	< 0.001
CEA	NA	NA	NA	0.898	2.128 (1.323 to 3.425)	0.002

model 1: based on clinicopathological characteristics alone

model 2: based on clinicopathological features and serum tumor markers

Abbreviations: *OR* odds ratio, *LVI* lymphovascular invasion, *SLN* sentinel lymph node, *CA* carbohydrate antigen, *CEA* carcinoembryonic antigen

### Performance of the pathology-based nomogram

#### Internal performance

**Fig. 3:**
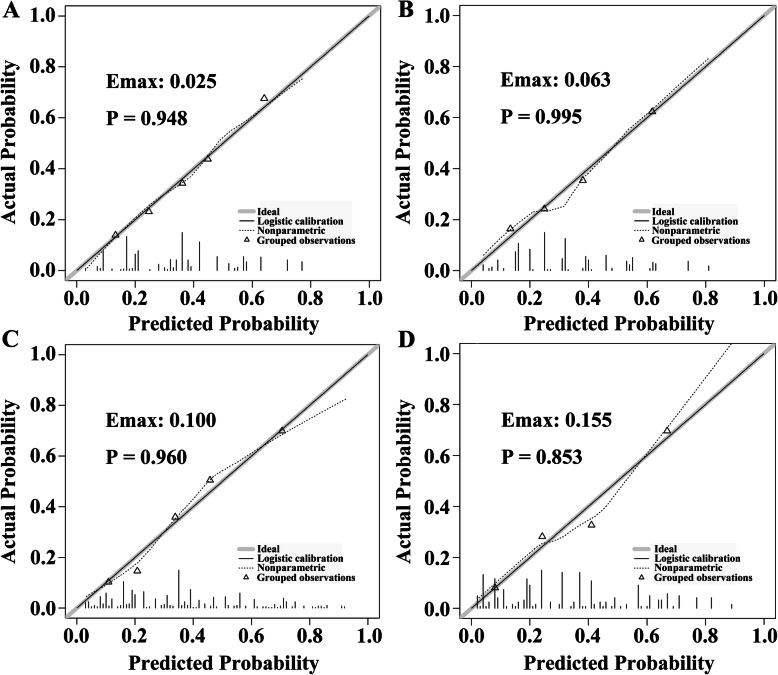
The calibration plot of the nomograms for the probability of axillary non-SLN metastases. **a** and **b** represent the calibration curve of pathology-based nomograms in the primary and validation cohorts, respectively; **c** and **d** represent the calibration curve of the combined nomograms in the primary and validation cohorts, respectively

**Fig. 4:**
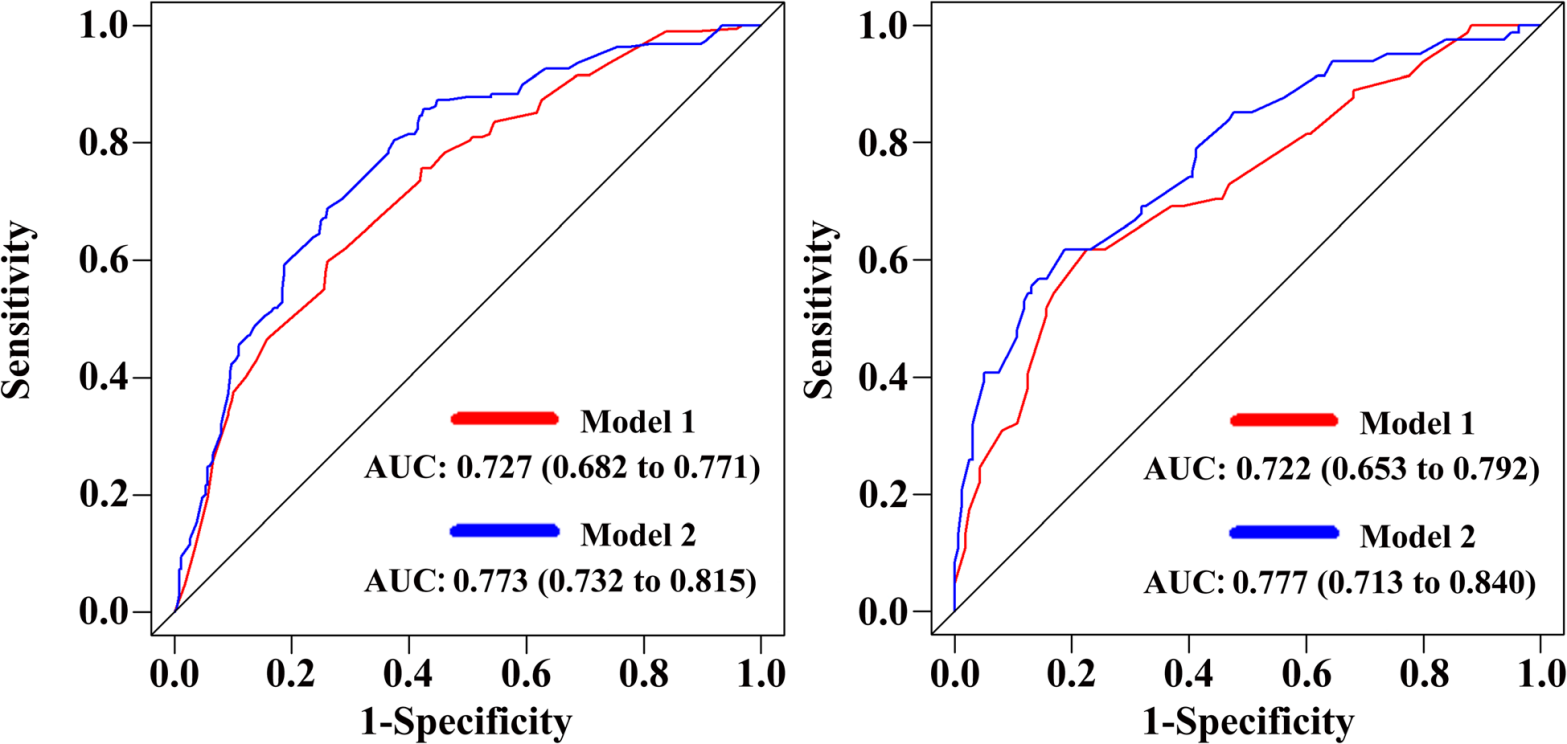
Receiver operating characteristic (ROC) curve based on the nomograms for non-SLN metastases. The red line represents the pathology-based nomogram. The blue line represents the combined nomogram. **a** and **b** represent discrimination ability of these two nomograms in the primary and validation cohorts, respectively

**Fig. 5:**
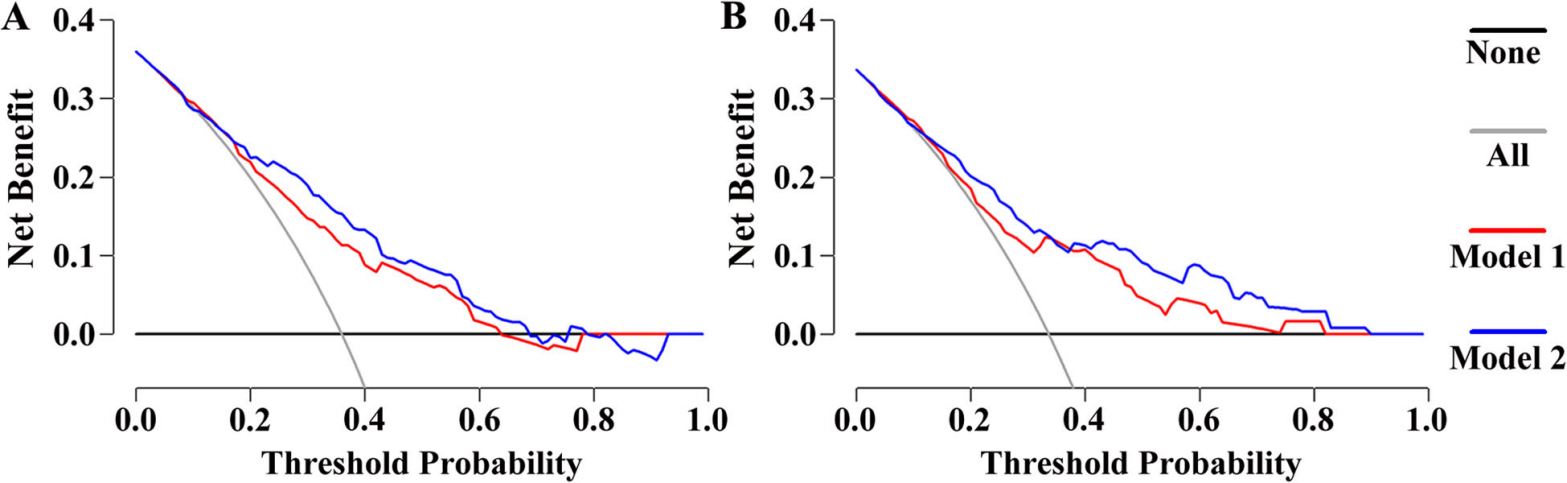
The decision curve analysis (DCA) for the nomogram in training and validation dataset. The red line represents the pathology-based nomogram. The blue line represents the combined nomogram. The grey line represents the assumption that all patients have axillary non-SLN metastases. The black line represents the assumption that none of patients have axillary non-SLN metastases. This graph gives expected net benefit of breast cancer patients with 1–2 positive sentinel lymph node using different clinical schemes. **a** and **b** represent the potential net benefits at different threshold probability in the primary and validation cohorts, respectively

The calibration curve of the nomogram showed good agreement between prediction and observation in the primary cohort (Fig. 3a). The Hosmer-Lemeshow test yielded a nonsignificant statistic (*P* = 0.948), which suggested that there was no departure from perfect fit. Besides, a strong discrimination ability with an AUC of 0.727 (95% CI: 0.682–0.771) was observed in the primary cohort (Fig. 4a). The decision curve revealed that if the threshold probability of a patient ranges from 0.09 to 0.64, using the nomogram to predict axillary non-SLN metastases would add more benefits than the assumption that all patients or none of patients had non-SLN metastases (Fig. 5a).

#### Independent validation

To determine whether the nomogram derived from the primary cohort was robust, we measured its performance in an independent validation cohort. The predictive score of each patient in the validation cohort was calculated by the regression coefficient to weight their respective predictors. In line with the results in the primary cohort, good calibration was also observed in the validation cohort with a nonsignificant statistic (*P* = 0.995) (Fig. 3b). In addition, the ROC curve yielded an AUC of 0.722 (95% CI: 0.653–0.792) (Fig. 4b) and the decision curve indicated more net benefits when the threshold probability ranges from 0.04 to 0.82 (Fig. 5b).

### Incremental predictive value of STMs for the pathology-based nomogram

To investigate the potential predictive value of STMs for axillary non-SLN metastases, the best subset regression was used to select the most useful indicators from three STMs (CA 125, CA 15–3 and CEA). Finally, CA 15–3 and CEA were included in the pathology-based nomogram and the nomogram was shown in Fig. 2b. The risk score in the combined model = − 1.168 + 1.034 (if multifocal tumor) + 0.655 (if high grade tumor (G2/G3)) + 0.866 (if lymphovascular invasion is positive) + 1.376 (if number of positive SLN = 2) + (0.750, if number of negative SLN = 1; 0.568, if number of negative SLN = 0). Predicted risk = 1/(1 + e^−risk score^). Calibration curves showed good agreement between prediction and observation in the primary (Fig. 3c, *P* = 0.960) and validation cohorts (Fig. 3d, *P* = 0.853). ROC analysis was further performed to compare the discrimination ability of the two nomograms. As shown in Fig. 4a and b, the nomogram integrating STMs and pathological features possessed a stronger predictive power for the non-SLN metastases in both the primary (0.773 (0.732–0.815) vs. 0.727 (0.682–0.771), *P* < 0.001) and validation cohorts (0.777 (0.713–0.840) vs. 0.722 (0.653–0.792), *P* < 0.001). Though there were several overlaps between both nomograms in decision curve, the addition of CA 15–3 and CEA brought more net benefits to the pathology-based nomogram within the threshold probability of 0.17–0.64 in both cohorts (Fig. 5a and b).

## Discussion

Using the data from 1150 early breast cancer patients in two independent cohorts, the findings of our study confirmed that pathology-based nomogram possessed a strong discrimination ability for axillary non-SLN metastases in Chinese breast cancer patients with 1–2 positive SLN. In addition, our study is the first to explore the predictive value of pathological features in combination with STMs for axillary non-SLN metastases. The results demonstrated that the addition of CEA and CA 15–3 could significantly improve the performance of pathology-based model in the primary cohort (AUC: 0.773 (0.732–0.815) vs. 0.727 (0.682–0.771), *P* < 0.001) and validation cohort (AUC: (0.777 (0.713–0.840) vs. 0.722 (0.653–0.792), *P* < 0.001). The information obtained in our study may greatly help clinicians to predict the risk of axillary non-SLN metastases and therefore to provide evidence to guide clinical decision-making of radiation field.

MSKCC nomogram based on eight pathological features, including number of tumor lesions, tumor size, tumor grade, number of positive SLN, number of negative SLN, detection methods of SLN, lymphovascular invasion and the status of ER, has been the most widely used model for predicting axillary non-SLN metastases [7]. However, its predictive value varies greatly among different populations. Degnim et al. reported that MSKCC nomogram possessed a strong discrimination ability with an AUC of 0.86 [8], but Klar et al. reported that its predictive value was only 0.58 [9]. The significant differences among different populations may be related to detection methods of SLN and evaluation criteria of pathological features.

The results of our study supported the conclusion that number of tumor lesions, tumor grade, lymphovascular invasion, number of positive SLN and number of negative SLN acted as an independent risk factor of axillary non-SLN metastases. Previous studies have reported that number of tumor lesions was significant associated with axillary non-SLN metastases, but not with SLN positive rate [16–18]. A possible explanation is that lymph containing tumor cells drained from multiple sites to the ipsilateral axillary, leading to a higher false negative rate of SLNB in the multifocal group than the unifocal group. Tumor with high grade [19, 20] and lymphovascular invasion [21, 22] has long been considered to be associated with non-SLN metastases due to its high aggressiveness. Number of positive SLN, number of negative SLN and the ratio of negative SLN to positive SLN has also been reported to be an independent predictor of axillary non-SLN metastases [7, 23]. However, tumor size and the status of ER was not found to be correlated with non-SLN metastases in our study. Chen et al. [24] and Abdessalam et al. [25] also reported that there is no significant correlation between tumor size and non-SLN metastases. Although several studies have reported the risk of axillary non-SLN metastases is higher in breast cancer patients with ER positive [26, 27], an increasing number of evidences suggested that there was no significant difference between them [28–30]. A possible reason is that included patients and evaluation methods of ER positivity are different among different institutions.

Although previous studies have demonstrated that preoperative STMs are important prognostic factors of breast cancer patients, the predictive value of STMs in combination with pathological features for axillary non-SLN metastases in patients with 1–2 positive SLN macro-metastases remains unknown [11, 12]. Li et al. reported preoperative serum CEA levels could be an independent prognostic factors for overall survival, and the nomograms including it would provide more personal forecasts information to optimize treatment for young breast cancer patients better [11]. Wang et al. reported that elevated serum CEA and CA 15–3 are significantly associated with bone metastases of breast cancer [31]. In line with these findings, the results of our study showed that breast cancer patients with positive axillary non-SLN are prone to have elevated serum CEA and CA 15–3. In addition, the performance of pathology-based model was significantly improved after the addition of CEA and CA 15–3.

## Conclusions

To the best of our knowledge, there are few studies that have assessed the validity of MSKCC breast nomogram in Chinese breast cancer patients. This research, therefore, overcomes this limitation to some extent. In addition, our study is the first to investigate the predictive value of STMs for axillary non-SLN metastasis. However, this study also has a few limitations. First, this study also has the inherent defects of retrospective and cross-sectional studies, such as patient inclusion and sample selection biases. Moreover, some features, such as imaging examination and some other detailed laboratory examination, were not well documented in our breast cancer database, which led to the exclusion of some potential predictors to ensure data authenticity and integrity.

## Data Availability

The dataset generated during the current study is available from the corresponding author on reasonable request.

## Abbreviations

SLN: Sentinel lymph node
ALND: Axillary lymph node dissection
ROC: Receiver operating characteristic
AUC: Area under the ROC curve
SLNB: Sentinel lymph node biopsy
STMs: Serum tumor markers
ER: Estrogen receptor
PR: Progesterone receptor
HER-2: Human epidermal growth factor receptor 2
CEA: Carcinoembryonic antigen
CA: Carbohydrate antigen
AIC: Akaike’s information criterion
